# Alterations in taste and smell associated with SARS-CoV-2: an exploratory study investigating food consumption and subsequent behavioural changes for those suffering from post-acute sequelae of COVID-19

**DOI:** 10.1017/jns.2022.19

**Published:** 2022-02-18

**Authors:** Yunus Khatri

**Affiliations:** Faculty of Environment, University of Leeds, Leeds, UK

**Keywords:** Post-acute sequelae of COVID-19, Anosmia, Ageusia, Altered eating, Food consumption, PASC, post-acute sequelae of COVID-19

## Abstract

*Objective*: To explore food consumption and subsequent behavioural changes amongst PASC suffers associated with alterations in taste and smell. *Design*: A qualitative study involving five focus groups. *Setting*: Birmingham and Leicester, England, United Kingdom. *Participants*: Forty-seven Post-Acute Sequelae of COVID-19 sufferers. *Results*: Shifts in taste and odour were very common with disgusting or unpleasant notes being perceived in many foods, including animal products rich in protein. Food consumption patterns varied affecting nutrition status, individuals weight, types of foods consumed, cooking habits, coping mechanisms, anxieties, family and social interactions. Individuals expressed the need to taste something or experience normal tastes and flavour. Low pH foods, highly processed foods which may contain large amounts of refined sugars as well as cold processed food were the preferred items for consumption. *Conclusion*: Olfactory dysfunction was related to the consumption of nutrients that require moderation and to the quality of life. Intervention at an early stage is necessary in order to help avoid such complications and thus, this work informs medical practitioners and health workers of the variety of food choices that are more acceptable for people suffering from altered tastes and odour perception.

## Introduction

COVID-19, caused by the SARS-CoV-2 virus, led to a pandemic in March 2020 and a global health crisis^(1,2)^. The clinical manifestations of COVID-19 are wide-ranging and non-specific varying from patient to patient. These symptoms may include but are not restricted to high temperature, sore throat, runny or stuffy nose, headache, dry cough, myalgia, dyspnoea, chest pain, joint pain, chills, loss of taste and smell, skin rash and discolouration of fingers and toes^(3,4)^.

Flavour is a complex multisystem percept experienced as a result of the combination of taste and odour^(5)^. Thus, flavour loss has a tremendous impact on eating quality of foods. Smell and taste dysfunction have been implicated in loss of appetite, unintended weight loss, malnutrition and reduced quality of life^(6)^. Alterations in taste and smell fall into two classes: loss of sensory acuity and distortions of taste and smell function. Dysfunction may be quantitative (hyposmia/anosmia) or qualitative (parosmia/phantosmia)^(7)^. Olfactory and gustatory dysfunction may exhibit to varying degrees as hyposmia (partial loss of smell), anosmia (total loss of smell), hypogeusia (decrease in taste sensitivity), ageusia (total loss of taste), hyposmia (decrease in sensitivity of odour), parosmia (altered sense of smell), phantosmia also termed olfactory hallucinations (smelling odours that are not there),^(8)^ phantogeusia also known as gustatory hallucinations (taste perceived in the oral cavity independent of any external stimuli)^(9)^ and chemesthesis (evoking sensations like burning, cooling and tingling). Anosmia and dysgeusia are often comorbid in COVID-19 patients^(10)^ with a significant association being displayed between both disorders^(11)^. Olfactory and gustatory disorders due to respiratory viral infections are not unknown^(12)^. Olfactory dysfunction as a sign of early infection indicating SARS-CoV-2 was promulgated in recent research^(11)^ with 77 % (95 % CI 61⋅4, 89⋅2 %) of patients identified having such impairment using objective methods in a meta-analysis^(13)^. Similarly, olfactory impairment is a highly reliable symptom and early sign^(14)^ in neurodegenerative diseases such as Parkinson's disease and Alzheimer's disease^(15,16)^ involving pathologic processes affecting the olfactory system prior to the onset of typical clinical manifestations. Hyposmia may be an early known pre-motor symptom in Parkinson's disease. Evidence mounted rapidly by scientists^(17,18)^ to include anosmia, hyposmia, dysgeusia and more recently phantogeusia^(19)^ as a novel finding in assessing patients with COVID-19. It was on 18 May 2020, when the British government announced through the press that the loss of taste and smell were officially added to the symptoms of COVID-19^(20)^, following recognition as crucial symptoms by the WHO.

The National Institute for Health Care Excellence (NICE)^(21)^ reports that recovery time is different for everyone but for many, symptoms are resolved within 12 weeks. For those experiencing extended symptoms, NICE^(21)^ defined long COVID, recently termed post-acute sequelae of COVID-19 (PASC), as ‘signs and symptoms that develop during or following an infection consistent with COVID-19, which continue for more than 12 weeks and are not explained by an alternative diagnosis’. A French study^(22)^ reported 24 % of patients had persistent taste and smell disorders 7 months after the onset of symptoms, while the National Institute for Health Research^(23)^ estimated approximately 10 % experience symptoms and impaired quality of life beyond 12 weeks. These figures show the gravity of the problem, with millions of people affected for extended periods of time, worldwide, and at the time of conducting this research, no recourse through clinical avenues. Fortunately, some countries such as the USA^(24)^ in 40 states, Canada^(25)^ and England^(26)^ have set up a number of clinics to help deal with problems associated with PASC. Owing to severe symptoms requiring urgent attention, priority is given to those with heart and respiratory problems, thrombo-embolic complications, cognitive blunting, fatigue, etc.^(27)^ with gustatory and olfactory dysfunction falling into a lower tier and only a few clinics attending to such dysfunction.

The aim of the present study was to explore food consumption and subsequent behavioural changes amongst PASC sufferers associated with alterations in taste and smell. To the best of the authors’ knowledge, a limited number of studies have been published regarding PASC in relation to food consumption and subsequent food-related behaviour. The quality of life studies concentrating on smell loss as well as working on both taste and odour loss^(28)^ from 9000 user-generated texts on the AbScent COVID-19 Smell and Taste Loss moderated Facebook support group have been conducted recently. Their findings revealed a number of altered eating-related aspects including the failure to consume food, loss of desire to eat food and engage socially, modification of body weight and altered relationship to self and others. Although results from this research concur with the aforementioned findings, greater detail is provided herein using focus groups of sufferers from PASC showing new evidence of different foods eliciting various odour and taste responses, the suitability of different foods, specific nutritional concerns, individuals’ ability (or inability) to cope with gustatory and olfactory changes as well as anxieties related to mental and emotional well-being.

## Methodology

The present study was conducted according to the guidelines laid down in the Declaration of Helsinki and all procedures involving human subjects/patients were approved by the Birmingham City University Ethics Committee, Department of Health Education and Life Sciences (Reference No. 8086). Written informed consent was obtained from all subjects/patients. Qualitative approaches are widely used to examine experience-centred designs^(29,30)^ with focus groups being emphasised as a preferred method^(31)^. This exploratory research was carried out between November 2020 and February 2021. Five focus groups were conducted: three in Birmingham and two in Leicester, England. A convenience sample method was adopted to recruit participants. Invitations to attend were sent, one per month for a period of 4 months, via the Health and Life Sciences website as well as circulating flyers with referrals being made from employees/students to individuals willing to participate in the present study. Eligibility criteria to participate included: (a) a minimum of 18 years of age, (b) tested positive for SARS-CoV-2 at least 3 months prior to this survey, (c) currently experienced alterations in taste and smell (d) willingness to participate within a week of registration. Excluded were those that reported any previous pre-existing olfactory/gustatory dysfunction (patients were asked: ‘Did you have an impairment in your sense of smell or taste prior to COVID-19 diagnosis?’). All individuals that met these specific criteria were included in the present study. Recruitment ended when an in-depth understanding of the alterations in taste and smell of food as well as subsequent behavioural changes were achieved. Furthermore, when results between groups showed no dis-similarity, saturation was reached and did not warrant additional sessions.

A total of forty-seven individuals participated in the present study. Individuals made contact with the lead researcher to register via email and arrange attendance at an appropriate session. Group sessions, held online using Microsoft Teams, were 60 min long. The facilitator followed a focus group guide with an icebreaker, introduction and eligibility criteria, aim, questions, discussion of topics and further probes. Each focus group discussion was recorded and transcribed, notes were taken on key issues arising from the discussion. Thematic analysis was conducted from the accumulated data and used to develop a common coding framework across the group discussions. Five themes were identified: occurrence and duration, foods implicated and flavour intensity, nutrient intake, coping and anxieties. Verbatim samples from recorded discussions are given below in quotation marks, presenting the diverse gustatory, olfactory and behaviour experiences on the topics raised. Below, some slang words were used, and to clarify, pee/piss refers to urine and crap refers to faeces.

## Results and discussion

In this cohort, individuals presented 117–199 d following a positive PCR confirmation of COVID-19 with smokers constituting 34⋅1 % of the sample. Table 1 shows the participant demographic profiles. Black, Asian and Minority Ethnicity (BAME) groups represented 63⋅8 % of the panellists. Geographical studies^(32,33)^ reported 43⋅1 and 49 % BAME classification in the cities of Birmingham and Leicester, respectively. This sample study reflects the participation and interest of the BAME community as individuals are at a higher risk of developing severe COVID and are four times more likely to die from the disease^(34–36)^. These communities are also frequently at higher risk for metabolic disorders including obesity, cardiovascular diseases, hypertension and type 2 diabetes^(37)^.

**Table 1.** Demographics of focus group panellists

Category	Count	Percentage
Age		
18–30	20	42⋅6
31–45	12	25⋅5
46–59	7	14⋅9
60–70	8	17
Gender		
Male	27	57⋅4
• Smokers	10	21⋅3
Female	20	42⋅6
• Smokers	6	12⋅8
Ethnicity		
White	17	36⋅2
BAME	30	63⋅8
Education (highest attained)		
High school	4	8⋅5
College	17	36⋅2
University	26	55⋅3
Employment status		
Unemployed	2	4⋅3
Student	25	53⋅2
Employed	9	19⋅1
Retired	11	23⋅4

The majority (53⋅2 %) of individuals were students as recruitment commenced within academic circles. There were 23⋅4 % of retired individuals as well as 17 % between the age of 60 and 70 years. Representation of this segment is important as an age-based vulnerability was uncovered for SARS-CoV-2, morbidity and mortality being skewed towards the elderly^(38)^.

Sensory distortions by patient dysfunction are shown in Table 2. The most prominent dysfunctions were hyposmia and parosmia at 63⋅8 and 59⋅6 %, respectively, while parageusia and anosmia were the least, exhibited at 14⋅9 and 19⋅1 %, respectively. The actual prevalence of chemosensory loss in self-reports is likely to be underestimated^(39)^, since many non-COVID-19 patients with such losses are unaware of their deficits until formal testing. In addition, a large representative cohort study^(40)^ found older black adults were more likely to lack awareness of olfactory dysfunction.

**Table 2.** Sensory distortions of patient dysfunctions

Condition	Number	Percentage
Ageusia	16	34⋅0
Parageusia	7	14⋅9
Phantogeusia	18	38⋅3
Anosmia	9	19⋅1
Hyposmia	30	63⋅8
Parosmia	28	59⋅6
Phantosmia	11	23⋅4

### Occurrence and duration

For most of the individuals (83 %), the first indication of COVID-19 occurred when individuals had no smell. Loss of taste followed after a few days followed by gustatory and olfactory perception being distorted for a duration of up to 6 months in some cases at the time that the present study was performed. Fluctuations in perceived odours were common in 7/28 individuals, and for some people who experienced PASC for at least 5 months, in particular, there was a shift in the perceived odorants. Altered tastes were perceived all over the mouth and not just the tongue.

*‘At first, I had no sense of smell and then 3 days later lost my sense of taste but after about three weeks everything just changed, my sense of smell became warped, I could smell pee and crap in my food. It was like being in a very unclean toilet. It's now six months and hasn't changed a bit.’*

*‘It just happened so quickly. My sense of smell went away completely and two days later everything was tasteless and then about four weeks later, I thought I was getting my taste and smell back but it was just so distorted. You get these strong disgusting smells of piss, crap and also chemical cleaner and smelly feet when you eat something and its lodged all over your mouth.’*

*‘Some foods was like pee and then changed to burnt rubber at about five months…improvement I'd say…ha, ha, ha!’*

Interestingly, abnormal odours were experienced continuously for eight individuals even without foods being consumed (olfactory hallucinations), while three non-smokers repeatedly experienced cigarette smoke sensations. When asked how they knew it was cigarette smoke, they answered ‘you get the smell just passing a smoker, we're all passive smokers’ and ‘it's everywhere, public transport, offices, you name it’.

*‘The smells I experience are not only when I am eating but constantly throughout the day. I can smell cigarette smoke for hours and I have never smoked.’*

### Foods implicated and flavour intensity

Taste (comprising of sweet, sour, salt, bitter and umami) on the tongue and odour in the nasal passages are perceived collectively as flavour^(5)^. However, twenty-two in this cohort related flavour to taste (when they actually meant flavour). Flavour profiling of foods in itself is very difficult to describe. A variety of different odours and intensities were noted by participants. Parosmia was evident in many cases (59⋅6 %) without any pleasant odours being perceived. Previous studies have also shown patients with parosmia reporting foul odours^(16,19,41)^. Animal products elicited responses that were perceived with disgust including faecal, urinary and ammoniacal odours arising from these food types which are known to be high in protein and rich in essential amino acids. The powerful odour stimulated emesis at times for two of the cohort. Product temperature plays a part in acceptance. A greater amount of odoriferous substances emanate when product temperatures are high.

*‘Meat, fish, milk and eggs are just like urine and faeces odours. Disgusting really.’*

*‘The intensity of the piss and ammonia smell increases as I chew meat and fish and I have vomited many times. The intensity will linger for maybe 30 seconds and fall away slowly, but not totally. I am gasping for breath and need to spit it out.’*

*‘Processed cold meats like chicken and turkey slices are the sort of things that are okay for me, but they must be cold. If any meat item is hot, I immediately get a bad smell.’*

Legumes, pulses and brown bread were identified as elastic or rubbery by eleven individuals (23⋅4 %). Tomatoes and mushrooms were perceived to be neutral, and white bread was much more palatable with no adverse responses from 13/47 (27⋅7 %) in the cohort.

*‘Beans, peas and lentils have an elastic band smell. I've tried them on their own and also in soup, no change in smell.’*

*‘Brown bread has an awful odour, it's like a mixture of burnt rubber and smelly feet. White bread has no taste, just bland so I prefer this, no problem with it.’*

*‘Tomatoes and mushrooms have no taste or smell at all. Flavourless!’*

A diminished umami taste could make these foods taste bland and less attractive^(42)^, explaining findings that support their hypothesis that umami is important for compliance with a healthy diet. Taste reduction of 25 %^(43)^ for umami is less frequently reported amongst COVID-19 sufferers.

Discussions ensued about fruits and vegetables and followed on to pickles, condiments and cheese. Fruits such as berries and stone fruits were the least pungent and had a mild metallic taste (six individuals). Highly acidic food items (low pH)^(44)^ were more acceptable to the palate of thirty-seven participants. Various lactic acid bacteria are used in pickles and cheese to produce lactic and acetic acid and are claimed to be probiotics^(45)^.

*‘Oranges, limes and lemons are great. They taste normal.’*

*‘Yeah, eating pickled foods such as gherkins, kim chi and sauerkraut. The sharp sour foods like citrus fruits…..ahm..lemons, limes, grapefruits are not offensive.’*

*‘Mature cheese, number 5, is sharp and at least I can taste it, Yoghurt has a sweet chemical taste.’*

The BAME community is known to consume lots of spiced foods^(46)^ but the results show that they (seven in the cohort) experienced difficulty in perceiving the various flavours. Furthermore, preferences for the consumption of fermented milk and vegetables (pickles, kimchi, etc.) are high^(46,47)^, providing distinct sharp flavours enhancing gustatory experience. However, this again was reported as diminished perception by the same seven individuals. Fermented meat products also contain lactic acid-producing bacteria that reduce the pH down to 5⋅3 or less. Commercial products contain sodium nitrite as a preservative and after drying may have a fat content between 40 and 50 %^(48)^. Coffee was perceived to have an odour of burnt tyres by 6⋅4 % of individuals but the flavour may be compounded by the bitter taste of water. Impaired chemesthesis^(43)^ was also evident as heat sensations from chillies were not noticeable by two individuals. The consumption of processed meats such as salami and pepperoni contain nitrites which are known to be carcinogenic^(49)^.

*‘Salami and pepperoni are what I eat now, I can eat them cold.’*

*‘Coffee tastes like burnt tyres.’*

*‘I normally eat lots of spicy foods, I can't even taste that. Not even the chillies.’*

*‘Water is bitter and the more I drink the more bitterness I experience at the back of my throat.’*

It is worth stating here that the NHS^(50)^ published information as part of symptom management including food items for those experiencing taste and flavour disorders suggesting the use of herbs and spices, opting for strong sharp foods as well as cold foods.

Results from a recent study^(16)^ examining olfactory hallucinations in Parkinson's disease patients found only a minority perceiving repulsive odours, while most of them (81⋅2 %) described more often a pleasant smell. Findings here are contrary to these results. These same authors also noted no differences in odour threshold, odour determination and odour identification between Parkinson's disease patients with and without parosmia and concluded that olfactory hallucinations are probably not related to the quantitative olfactory dysfunction leading to hyposmia/anosmia, but rather to other mechanisms.

### Nutrient intake

It is well reported that losses of taste and smell can exacerbate poor nutritional status by reducing oral intake and interfering with cephalic phase reflexes necessary for optimal nutrient absorption and digestion^(51)^. Fluitman^(42)^ found that self-reported poor taste was associated with poor nutrition but no other loss of smell and taste score was associated with poor appetite or poor nutrition. From this cohort, thirteen individuals ate foods only when absolutely hungry. Participants (18/47, 38⋅3 %) revealed that there was an inordinate desire to experience taste, odours and flavour to satisfy previous experiences of normality.

‘*I just cannot eat anything. Yuk, I only eat when I have to.’*

*‘In a way I am happy I am losing weight, that's good but I'm a student studying Nutrition and I know if this continues, I will be malnourished. Because I'm vomiting, I decided to take supplements and protein powders that my brother uses for body building.’*

*‘I am so hungry all the time and I cannot eat meat any more at all. The vegetarian and vegan meals also taste of a mixture of burnt rubber and chemicals. I have lots of junk food now just for a flavour hit… things like Coke, Victoria sponge cakes with jam, biscuits and chocolate coated doughnuts. I'm putting five teaspoons of sugar in my coffee, used to put two before, so I am able to taste something. I know some people here have said that they have lost weight but I have put on weight and lots of it.’*

*‘Me too, but I am borderline diabetic I think I will become a diabetic very soon with all the Cokes, cakes and biscuits everyday.’*

*‘The previous chap mentioned eating sugary stuff, for me its salt. I add tonnes of it as there is just no taste. I am quite happy with salami and peperoni though. I need to taste something.’*

*‘Not getting my five a day and not following the Eatwell Guide is really worrying.’*

Guidance from the British Dietetic Association (BDA)^(52)^ recommends consumption of a limit of 30 g sugar, 6 g of salt and a reference intake of 70 g fat (20 g being saturated) and 90 g of total sugars (reference intake for total sugars includes sugars from milk, fruits and vegetables, as well as added sugar) per day. A regular can of cola contains 35 g sugar, equivalent to six teaspoons of sugar. Due to a protracted inability to perceive any taste or flavour, a number of participants (34 %) resorted to consuming higher than normal amounts of sugar, salt, fat and highly processed foods [defined in the report by the European Prospective Investigation into Cancer (EPIC)^(49)^ to include foods that are industrially prepared or with such ingredients under specific categories]. The risks associated with such food consumption behavioural patterns given above, leading to type 2 diabetes, hypertension, obesity and cardiovascular disease, are well documented^(53-55)^. In addition, concern was expressed about the lack of adequate nutrient intake by 4/47 (8⋅51 %) in the cohort.

The author has used the term higher than normal in light of the fact that participants self-reported terms such as ‘lots of’ and ‘tonnes of’ in the verbatims. The Eatwell Guide published by Public Health England^(56)^ is extremely difficult to follow for those stricken by PASC. The foods that are being ingested should be part of a calorie-controlled diet. However, this is a testament to the fact that a healthy eating regime is also tied to taste and flavour perception. Supplements and formulated drinks are being considered by three people in the groups as this may assist in remedying the lack of sufficient nutrient consumption. The Eatwell Guide recommends eating small amounts of foods high in fat, salt and sugar and less often.

New research findings^(53)^ have revealed that olfactory dysfunction is related to the selection of higher energy density foods and poorer diet quality, as well as lower consumption of foods that constitute an adequate diet (e.g., fruits, vegetable and whole grains) and larger consumption of nutrients that require moderation (e.g., added sugars and saturated fats). The same authors explained from the data that there is a tendency to compensate for chronic olfactory dysfunction by seeking positive primary taste qualities (sweet and salty) and the positive texture components of fat, which could in turn explain the increased risk of weight gain^(57,58)^ and cardiovascular disease risk^(59)^ among those with olfactory dysfunction.

### Coping

In an attempt to minimise the sensorial effect of gustatory and olfactory dysfunction, some members of the groups (12⋅8 %) utilised methods that decrease residence times in the buccal cavity. This has been done by reducing particle size so that there is very little need to chew the food or it may be homogenised and drawn with a straw. The majority of individuals (26/47, 55⋅3 %) presented the view that they were forcing themselves to eat. Furthermore, by obstructing the nasal passage and preventing the flow of air towards the olfactory region, three individuals were able to reduce the unpleasant odours allowing them to consume a little more food, but not for long.

*‘Pulverised materials are the only way for me to consume the foods through a straw and then just swallow. Force feeding myself cause I'm hungry.’*

*‘I eat my food in small bites so that I don't need to chew much.’*

*‘I have held my nose and yes it's possible to take a few mouthfuls, but not for long. I could not keep my nose blocked for say… even one minute.’*

### Anxieties

The impact on individuals’ quality of life has been vast with adverse effects on mental and emotional well-being. No instruments were used in the present study to evaluate anxiety or psychiatric symptoms. In dialogues, they were very annoyed at what has become of them. Not being able to eat normally has left individuals in despair, lacking in hope, unsocial, unaccepting of others eating with them and also separated from the family unit at the table. One particular quote shows aspects of an overbearingly tense episode at the table leading to breathlessness and food being expelled from their mouths. One of the panellists was in tears as she was unable to cook and pass on her traditional recipes and techniques down to her granddaughter. Cooking has been a central theme in human evolution^(60)^ and sharing food at mealtimes defined human social culture since hunter-gather times^(61)^. Mealtimes have fragmented families as 8⋅5 % of participants could not endure eating with others and isolated themselves. Disruption has only meant disarray for families and an adjustment to lifestyles.

*‘When I hear people eating crisps and crackers, that sound,….. I am battling within myself and angry as I can't eat these things and want to tell them to stop eating in front of me. I really get frustrated and just leave the room.’*

*‘I used to love food, now I hate it (in tears). Even when I am cooking, I can smell urine. I can't cook anything or teach my granddaughter how to cook anymore.’*

*‘My mouth has a sort of burnt rubber taste constantly. I don't want to open my mouth because someone may think my mouth stinks.’*

*‘Everything tastes revolting. I just want to this to end.’*

*‘I can smell dirty socks all the time. I have tried a nose cleanse, would you believe, that I read about and it doesn't work.’*

*‘When my sense of smell first became warped, I would retch on the table and had difficulty breathing and two of my family members vomited seeing this. I decided to eat separately from them now. We don't eat together anymore.’*

*‘I am losing a lot of weight and if this keeps on going I will need to see a doctor, but they don't see us face to face. They should actually see how much weight I've lost. I'm just depressed about this whole thing and no access to doctors.’*

The exact mechanism of olfactory and gustatory loss is complex and not fully understood. A number of papers have hypothesised sensory neuron attack as well as co-expression of angiotensin-converting enzyme 2 (ACE2) and transmembrane serine protease 2 (TMPRSS2) as the chief cause of olfactory–gustatory disorders, while the competitive activity of COVID-19 on ACE2 receptors in the taste buds as well as sialic acid receptors^(10,12,62)^ affecting taste perception. Raised cytokine levels in COVID-19 patients were found in the olfactory epithelium signifying inflammation of the olfactory tract may have a direct role in the progression of sensory loss. At present, the causal relationship between COVID-19 and the development of Parkinson's disease lacks robust clinical/etiopathogenic evidence^(19)^.

Anxieties were expressed as there was a need to physically present to a doctor for treatment and this cannot be over-emphasised. All the participants have patiently waited and had not been seen by a doctor as yet (only telephone consultations). Moreover, health practitioners tend to overlook olfactory dysfunction^(28)^ leaving individuals without recourse. PASC clinics may offer therapies for olfactory-taste disorders including olfactory training, intranasal application of sodium citrate and vitamin A, corticosteroids, caffeine, theophylline, omega-3 fatty acids as well as systemic use of α-lipoic acid and zinc sulphate; however, their effectiveness remains to be proven by means of randomised clinical trials as some results have been contradictory^(62)^.

## Study limitations

The present study had a number of limitations. The work relied on forty-seven participants self-reporting COVID-19 symptoms beyond 12 weeks without a control group. No specific questionnaire was used to test patient anxieties and psychiatric symptoms. Limitations also include the lack of any objective or standardised measurements of health, dietary intake, nutritional status, medication use, physical activity and anthropometrics. Given the circumstances, it was not possible to recruit more individuals since they were to confirm that they had PASC taste and smell disorders for at least 3 months. No physical proof of date for the COVID-19 positive test was requested. However, in view of the discussions and evidence from previous studies, there is little to indicate that they were not infected with COVID-19 and experiencing symptoms for extended periods of time.

## Conclusion

Loss of taste and/or smell can affect nutritive intake and quality of life. Further quantitative research is required to investigate the actual nutrient intake from the spectrum of foods consumed in PASC patients. It is conceivable that around the globe, millions of people suffering from PASC with varying taste and smell disorders face the burden of an uncertain future from a health and eating perspective. Early intervention is necessary in order to assist those individuals stricken by protracted COVID-19 and their food consumption practices. The consequences of such dietary intake increase the risks associated with poor dietary practice. This work increases the knowledge of options for the different foods that are more acceptable for people that suffer from altered tastes and odour perception. It provides a greater choice of alternatives to make informed decisions in order to reduce the overwhelming pungency of foods that restrict suitable nutritive intake. Rather than play the issues relating to olfactory and gustatory dysfunction down, there is a need to relieve the plight of PASC sufferers so that they can return to their own normality.
